# Primary Patient-Derived Cancer Cells and Their Potential for Personalized Cancer Patient Care

**DOI:** 10.1016/j.celrep.2017.11.051

**Published:** 2017-12-12

**Authors:** David P. Kodack, Anna F. Farago, Anahita Dastur, Matthew A. Held, Leila Dardaei, Luc Friboulet, Friedrich von Flotow, Leah J. Damon, Dana Lee, Melissa Parks, Richard Dicecca, Max Greenberg, Krystina E. Kattermann, Amanda K. Riley, Florian J. Fintelmann, Coleen Rizzo, Zofia Piotrowska, Alice T. Shaw, Justin F. Gainor, Lecia V. Sequist, Matthew J. Niederst, Jeffrey A. Engelman, Cyril H. Benes

**Affiliations:** 1 Massachusetts General Hospital Cancer Center, Boston, MA 02129, USA

**Keywords:** patient-derived cancer cells, NSCLC, personalized medicine

## Abstract

Personalized cancer therapy is based on a patient’s tumor lineage, histopathology, expression analyses, and/or tumor DNA or RNA analysis. Here, we aim to develop an *in vitro* functional assay of a patient’s living cancer cells that could complement these approaches. We present methods for developing cell cultures from tumor biopsies and identify the types of samples and culture conditions associated with higher efficiency of model establishment. Toward the application of patient-derived cell cultures for personalized care, we established an immunofluorescence-based functional assay that quantifies cancer cell responses to targeted therapy in mixed cell cultures. Assaying patient-derived lung cancer cultures with this method showed promise in modeling patient response for diagnostic use. This platform should allow for the development of co-clinical trial studies to prospectively test the value of drug profiling on tumor-biopsy-derived cultures to direct patient care.

## Highlights

• Successful culture of cancer cells from tumor biopsies
• An immunofluorescence-based assay to quantify drug sensitivity in mixed cell cultures
• NSCLC patients’ biopsy culture sensitivities reflect clinical response

## Graphical Abstract

**Figure:**
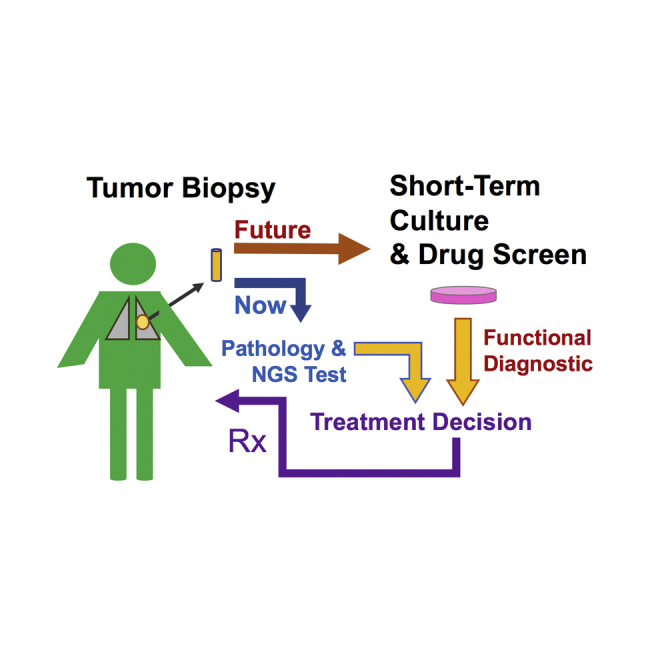

## Teaser

Kodack et al. report on the development of cancer models from tumor biopsies and technologies toward a functional approach for personalized medicine. They describe the ability to reliably test drug response in patient-derived samples of mixed cell populations. In doing so, they show that patient biopsy cultures may predict patient clinical responses.

## Introduction

Identification of somatic genetic driver alterations in tumors can direct selection of effective targeted therapies. While advances in sequencing technology and target identification have had a major impact, only a small fraction of cancer patients are treated based on the identification of specific genetic mutations (Dienstmann et al., 2015). Furthermore, responses to targeted therapies among genetically defined patients are heterogeneous. Matching therapeutics to genetic mutations is currently limited by an incomplete understanding of the relationship between tumor genotype and drug sensitivity. Moreover, the opportunity represented by rare, exceptional responders in unselected patients is not exploited by current patient selection strategies.

Functional testing of living cancer cells derived from patient biopsies may be able to overcome the limitation of predicting a cancer’s phenotype based on solely its genetics (Friedman et al., 2015). Leukemia and other hematological malignancies offer a tractable clinical avenue for functional testing due to the relative ease of obtaining a large number of viable cancer cells. Several groups have reported high-throughput clinically translatable approaches to measure leukemic responses *ex vivo* and, in some cases, correlated them to patient responses (Pemovska et al., 2013, Tyner et al., 2013). Importantly, based on these early successes, at least one phase-2 clinical trial in relapsed AML has been initiated to test the clinical utility of this approach (clinicaltrials.gov identifier NCT: NCT01620216).

Historically, however, culturing cancer cells from solid tumors has generally not been rapid or readily feasible. Adding to this challenge, patients presenting with metastatic disease often undergo a diagnostic needle biopsy rather than surgical resection, and the biopsy material may be relatively scant. Recent work by Dr. Richard Schlegel and colleagues (Liu et al., 2012, Liu et al., 2017, Palechor-Ceron et al., 2013, Suprynowicz et al., 2012) has established conditions that allow for more robust and, at times, otherwise unattainable efficiency in culturing cancer cells from surgical or biopsy samples. Schlegel and colleagues reported “conditional reprogramming” as a method to generate cell cultures from normal and tumorous recurrent respiratory papillomatosis that were then tested for chemosensitivity (Yuan et al., 2012). The chemotherapy identified to be most effective *in vitro* produced a durable cytostatic effect in the patient.

We previously described a pharmacogenomic approach to identify therapeutic strategies that overcome resistance to targeted therapies using cancer cells derived from biopsies of resistant non-small-cell lung cancer patients (Crystal et al., 2014). In this study, focused on a limited number of samples, we demonstrated that pharmacological screening could identify genetic mechanisms of resistance that were present (and not necessarily identified prior to the screen) as well as non-genetic mechanisms of resistance. Furthermore, targeting *in vitro* functional resistance mechanisms yielded bona fide tumor regressions *in vivo* in 5 out of 5 cases. Development of additional models across diverse clinical trials continues to provide a unique opportunity to define resistance mechanisms and therapeutic options. These experiments were performed using pure cancer cell populations that took, in most cases, more than 6 months to develop, precluding their use to impact the care of the biopsied patient in a timely manner. However, in order to impact individual patient care, an important goal of our research is to develop a methodology capable of testing cancer cell response within weeks of the biopsy. Therefore, we aimed to develop an assay to analyze a high-throughput pharmacological screen using biopsy cultures of mixed cell populations—cancer and stromal cells of the tumor as well as feeder fibroblasts—growing in defined media. In doing so, we suggest a novel functional diagnostic assay that could be used to examine the utility of functional testing, in addition to genetic sequencing, to match therapies to individual cancer patients.

## Results

### Generation of Primary Cancer Cells from Patient Tissues

As of June 2016, we attempted to generate patient-derived cultures from 568 patient specimens, including core biopsies, fine-needle aspirates, pleural effusions, resections, or autopsy specimens. The samples included a variety of malignancies, including lung, breast, colorectal, endometrial, pancreatic, and head and neck cancers. The success rate of developing a finished cancer cell monoculture for each tumor type is summarized in Table 1. A finished culture is one in which the cancer cells no longer require an irradiated fibroblast feeder layer for growth; are free of stromal fibroblasts (as visualized by eye); can be cryopreserved, thawed, and re-grown; and share the same driver mutation(s) as the initial biopsy specimen. A failed culture exhibits no cancer cells after 6 months of culture. Using these criteria, the success rate in generating a cancer cell monoculture across all cancer types was 26%. The vast majority of samples were lung cancer (373), and we were successful in generating a finished cancer cell line in 29% of these cases. While we were not powered to statistically compare success rates of cancer cell-line generation across all tumor types, there was a statistically significant higher success rate in establishing a pure cancer cell culture from lung cancers compared to breast cancers (29% versus 15%; p < 0.01). The success rates among luminal and basal breast cancers are detailed in Table S1.

**Table 1:** Success of Cancer Cell Line Generation by Tumor Type

Tumor Type	Number Finished	Number Failed	Total Processed	Percent Successful
Breast	16	88	104	15
Colorectal	5	15	20	25
Endometrial	1	10	11	9
Head and neck/salivary gland/oropharyngeal/ squamous cell carcinoma	2	9	11	18
Lung	109	264	373	29
Melanoma	1	6	7	14
Pancreatic/gallbladder	6	10	16	38
Thyroid	4	10	14	29
Unknown	4	8	12	33
Total	148	420	568	26

See also Table S1.

We retrospectively reviewed 286 lung cancer samples to investigate why the majority of our samples failed to generate cancer cell lines. The overwhelming reason for failure was the lack of cancer cells at the initiation of cell-line development, as identified by visual inspection of cells under the microscope immediately after tissue dissociation (Table S2). Another reason for cell-line failure was the outgrowth of stromal cells, primarily stromal fibroblasts. As we gained experience in the culture process, we used a number of techniques to minimize stromal fibroblast outgrowth. We took advantage of the fact that, in some cases, the fibroblasts detached faster and adhered more slowly than the cancer cells of epithelial origin, and we used differential trypsinization timing and re-adhesion to separate cancer cells from fibroblasts. In addition, cancer cell colonies were sometimes specifically picked from the plate to separate them from the stromal fibroblasts. Furthermore, we used anti-fibroblast columns to separate fibroblasts from the rest of the culture.

We received lung cancer samples of different types, including core biopsies, fine-needle aspirates, pleural effusions, resections, and autopsy specimens. We retrospectively examined the success across these differing sample types (Table S3). Cancer cell cultures were derived from pleural effusions at a significantly higher rate compared to core biopsies (42% versus 23%; p < 0.001). Several factors might contribute to this difference, including the enzymatic dissociation needed for core biopsies but not pleural effusion, the higher number of cancer cells in pleural effusions as estimated by visual inspection, the likely presence of fewer fibroblasts in pleural effusions, and/or the biology of the cancer cells. There was not a statistically significant difference in the success rates among the other sample types, besides the comparison of pleural effusions to autopsy samples (42% versus 13%; p < 0.01). The success rate of autopsy samples was likely low because of a combination of poor tissue viability and an increase in bacterial and fungal contamination. Some of the lung cancer biopsies came from metastatic sites outside the lung, including liver, lymph node, and bone. We examined the success rate of cell-line generation across different biopsy sites. While liver biopsies trended to be more successful than lung biopsies (36% versus 25%), this difference was not statistically significant (Table S4). Lymph node biopsies were successful 14% of the time, and the small number of bone biopsies (N = 2) did not allow for meaningful conclusions. Thus, we were able to culture cancer cells from lung cancer taken from different primary or metastatic sites, including lymph nodes.

While our initial culture conditions consisted of standard growth media, including RPMI, DMEM, and ACL4 with fetal bovine serum (FBS), on collagen-I-coated dishes, we later adopted approaches pioneered for the culture of primary human embryonic stem cells, human keratinocytes, and human non-keratinocyte epithelial cells (Chapman et al., 2010, Terunuma et al., 2010, Watanabe et al., 2007). This culture method contained irradiated fibroblast feeder cells and defined media: a 3:1 ratio of Ham’s nutrient mixture F-12:DMEM, FBS, hydrocortisone, epidermal growth factor (EGF), insulin, cholera toxin, adenine, and a Rho-associated protein kinase (ROCK) inhibitor. We termed this culture method feeder+TCM (tumor culture media) and refer to it as such throughout this article. In some cases, a single sample was cultured in two or more media conditions at the time of initial plating; therefore, the importance of the media in cell-line generation could be evaluated more directly. We compared the success and failure rates within 61 lung cancer samples in which a cell line was established in at least one culture condition and was initially plated in more than one condition. Table S5 illustrates the success rate of using the irradiated human foreskin feeder cells and TCM (feeder+TCM) compared to all other media, including RPMI and 10% FBS (R10), DMEM and 10% FBS (D10), ACL4 and <5% FBS (A < 5), and ACL4 and ≥5% FBS (A ≥ 5), within each tissue type. In 46% of the biopsy tissues, the feeder+TCM culture was the only condition successful in generating a cancer cell line. Both feeder+TCM and any other type were successful in nearly a third of the biopsies that produced a cell line, and a culture condition other than feeder+TCM was uniquely successful in less than a quarter of them. Therefore, when a cell line was successfully developed from a needle biopsy, the feeder+TCM had an overall success rate of 77%, compared to 54% for the other media types combined. In 35% of pleural effusions, feeder+TCM culture was the only successful condition, compared to a 19% success rate for all other media types. Pleural effusions were successful in both feeder+TCM and any other media type 46% of the time. Therefore, for pleural effusions, feeder+TCM had an overall success rate of 81% for generating a cell line, compared to 65% success for all other tested media combined. While some trends can be observed, these numbers were insufficient to yield statistically meaningful analysis. In summary, in our hands, working mostly with non-small-cell lung cancer (NSCLC) samples (core biopsies and pleural effusions), the feeder+TCM culture condition tended to be superior to all other media in generating a cancer cell line, and media type was more impactful for core biopsies than pleural effusions.

### A Miniaturized Immunofluorescence-Based Assay to Drug Screen Mixed Cell Populations of Patient-Derived Cancer Models *In Vitro*

Since the ultimate goal of this effort is to directly impact patient care, there is a need to interrogate biopsy cultures as quickly as possible. The initial biopsy culture generally contains multiple cell types, including fibroblasts and lymphocytes, and a variable number of cancer cells. Additionally, the addition of an irradiated fibroblast feeder layer tended to increase the likelihood of a successful biopsy culture and increased the growth rate of the culture (not statistically significant at the current sample size). Therefore, we aimed to develop a method to perform pharmacological screening of biopsy cultures using limited numbers of cancer cells that co-exist with stromal cells. Standard cell-viability assays that measure bulk culture viability, including CellTiter-Glo and MTT, do not discriminate between different cell populations within the same well. Therefore, we developed an immunofluorescence-based cell scoring method to specifically quantify cancer cell number. This method seeks to overcome the following three obstacles: (1) patient biopsies contain a small number of cancer cells; (2) culture development is potentially more successful on an irradiated fibroblast feeder layer, which confounds cell viability assessment; and (3) noncancerous cells, particularly fibroblasts, often survive during initial culture expansion. The presence of stromal cells—in particular, carcinoma-associated fibroblasts—in the biopsy culture may allow for a more representative assessment of the patient’s tumor. Indeed, fibroblasts and the growth factors they produce have been identified to mediate resistance to targeted therapies (Capparelli et al., 2015, Straussman et al., 2012, Wilson et al., 2012). Finally, due to the difficulties of establishing a pure cancer cell line, the ability to study drug response in mixed cell cultures, prior to the establishment of a pure cell line, may allow for an increased number of clinically relevant testable samples—both due to time and overall success.

To identify epithelial cancer cells within mixed cell populations, we screened antibodies used in clinical pathology that can reliably identify cells of epithelial origin (Table S6). We identified a cocktail of two monoclonal antibodies, one against cytokeratin 8 and another against cytokeratin 18, as the most consistent identifier of epithelial cancer cells via immunofluorescence. This exact antibody cocktail is used in pathology (on formalin-fixed paraffin-embedded samples) for the identification of epithelial tumors, since CK8 and CK18 are expressed in nearly all carcinomas of epithelial origin (Moll et al., 2008). Not only was the CK8/18 cocktail consistent in its staining of epithelial cancer cells, it also recognized cancer cells that had undergone an epithelial-to-mesenchymal transition (EMT) morphological phenotype (a clinically observed resistance phenotype; indicated by asterisks in Table S6). Importantly, the CK8/18 cocktail did not stain human fibroblasts. In addition to lung adenocarcinoma cells, the CK8/18 cocktail also recognized squamous cell lung cancer, small-cell lung cancer, and breast, bladder, intestinal, colorectal, and pancreatic cancer cells (Tables S6 and S7). Of note, while still identifiable, melanoma cells were only weakly stained.

Co-staining of CK8/18 with the nuclear (DNA intercalant) marker Hoechst 33342 allowed for the identification, and quantification, of two types of cells, CK8/18-negative and CK8/18-positive, corresponding to non-cancer and cancer cells, respectively. Figure S1 shows a primary patient tumor culture with mixed cell populations growing in a single well of a 384-well plate. Hoechst 33342 staining is indicated in blue (Figure S1A) and CK8/18-Alexa647 as green (Figure S1B). Cell imaging was performed using Molecular Devices’ ImageXpress Micro XL high-content imager. Molecular Devices’ MetaXpress software was used to score all nuclei (Figure S1C, white) and CK8/18-positive cytoplasm (Figure S1D, red). The overlay of the true nuclei scored and the CK8/18-positive cells scored is shown in Figure S1E; this scoring is used to quantify CK8/18-positive (red) and CK8/18-negative nuclei (white).

**Figure 1:**
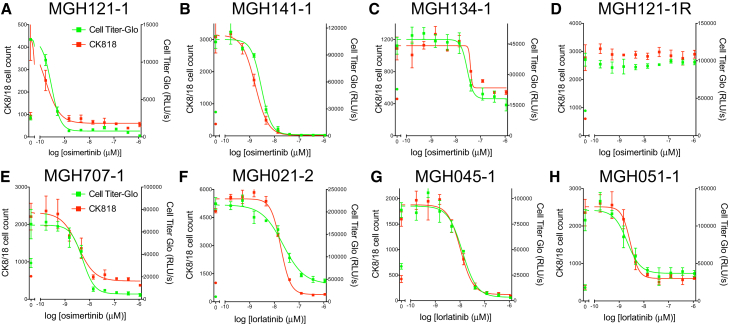
Comparison of Responses to EGFR and ALK Inhibitors Measured Using CK8/18-Positive Cell Count and CellTiter-Glo in *EGFR*-Mutant and *ALK*-Translocated Cancer Cells (A–H) Patient-derived *EGFR*-mutant (A–E) or *ALK*-translocated (F–H) lung cancer cells growing in 384-well plates in R10 or D10 media were treated with nine doses of the EGFR inhibitor osimertinib or the ALK inhibitor lorlatinib for 4–7 days. (A) MGH121-1. (B) MGH141-1. (C) MGH134-1. (D) MGH121-1R. (E) MGH707-1. (F) MGH021-2. (G) MGH045-1. (H) MGH051-1. (MGH121-1R cells were made resistant *in vitro* to the third-generation EGFR inhibitor WZ4002 by increasing its concentration over time.) Plates were either fixed and stained with Hoechst 33342 and the anti-CK8/18 antibody to determine the change in CK8/18-positive cells (red curves, left y-axes) or treated with CellTiter-Glo to determine the change in ATP concentration (green curves, right y-axes). Circles indicate CK8/18-positive cell number (red) or CellTiter-Glo measurement (green) at the day of treatment initiation. Nonlinear regression curves fit to the data points are shown, and data are represented as mean ± SD with n = 4 replicates and experiments performed twice.

We first examined the ability of the immunofluorescence assay to replicate dose-response curves obtained with a broadly used bulk cell population viability reagent, CellTiter-Glo. We plated the established (pure cancer cell) cell lines of *EGFR* mutant or *ALK*-translocated NSCLCs into 384-well plates and compared their sensitivity to an EGFR or ALK inhibitor, respectively, using CellTiter-Glo or the immunofluorescence-based method (Figure 1). We found that the immunofluorescence assay (red curves) replicated the Cell Titer-Glo (green curves) results very well when comparing these pure cancer cell populations. These findings were consistent across cells with a variety of sensitivities to EGFR or ALK inhibition, including those with high sensitivity, moderate sensitivity, or no sensitivity. Of note, the assay remained robust when as few as 100 cells were plated into a single well of a 384-well plate, illustrating the ability of the assay to determine response with a small number of cells. Additionally, the immunofluorescence-based viability assay can be performed and analyzed within 48 hr, allowing for rapid acquisition of data.

**Figure 2:**
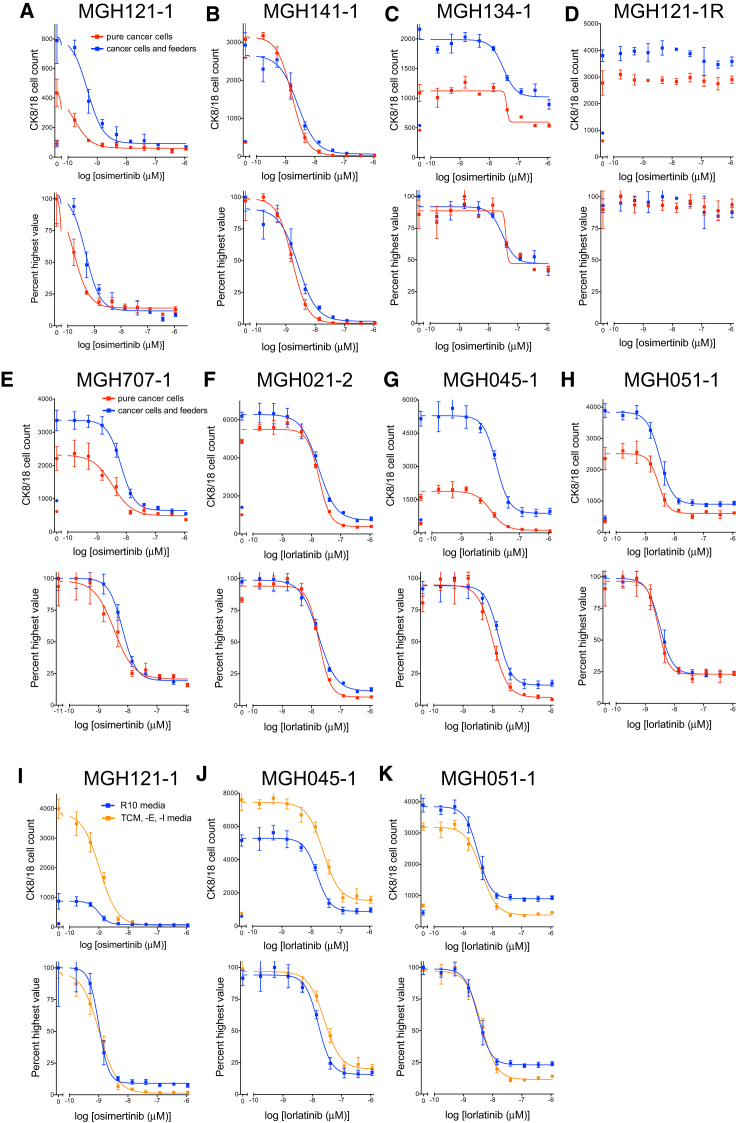
Sensitivity of *EGFR* Mutant and *ALK*-Translocated Lung Cancer Cells to EGFR or ALK Inhibitors, Respectively, Growing Alone or in the Presence of Irradiated Human Foreskin Fibroblasts and in R10 or TCM, -E, -I Media (A–H) Patient-derived *EGFR*-mutant (A–E) or *ALK*-translocated (F–H) lung cancer cells growing alone (red curves) or in the presence of irradiated human foreskin fibroblasts (blue curves) in 384-well plates in R10 or D10 media were treated with nine doses of the EGFR inhibitor osimertinib or the ALK inhibitor lorlatinib for 4–7 days. (A) MGH121-1. (B) MGH141-1. (C) MGH134-1. (D) MGH121-1R. (E) MGH707-1. (F) MGH021-2. (G) MGH045-1. (H) MGH051-1. (MGH121-1R cells were made resistant *in vitro* to the third-generation EGFR inhibitor WZ4002 by increasing its concentration over time.) (I–K) Patient-derived *EGFR*-mutant (I, MGH121-1) or *ALK*-translocated (J and K, MGH045-1 and MGH051-1, respectively) lung cancer cells growing in R10 media (blue curves) or in TCM, -E, -I media (orange curves) in the presence of irradiated feeder fibroblasts in 384-well plates were treated with nine doses of the EGFR inhibitor osimertinib or the ALK inhibitor lorlatinib for 4–7 days. Plates were fixed and stained with Hoechst 33342 and the anti-CK8/18 antibody to determine the change in CK8/18-positive cells. Raw CK8/18 cell counts are illustrated on the top graphs, and normalized values (percent highest) are depicted on the bottom. Circles indicate CK8/18-positive cell number at day of treatment initiation. Nonlinear regression curves fit to the data points are shown, and data are represented as mean ± SD with n = 4 replicates and experiments performed twice. See also Figure S2.

We next tested the ability of the assay to perform in co-culture conditions that more closely mimic the early culture of a patient’s biopsy. We mixed established cancer cells with irradiated human foreskin feeder fibroblasts, the same cells used for the initial culture of patient samples (Figures 2A–2H). This allowed us to ask two questions: (1) could the assay perform adequately in mixed cultures, and (2) does the presence of the irradiated foreskin fibroblasts impact sensitivity to therapies? Analysis of co-cultures of cancer cells and irradiated feeder fibroblasts (Figures 2A–2H, blue curves) illustrated a dose-response curve similar to that of pure cancer cell cultures (Figures 2A–2H, red curves). These data show that (1) the assay distinguished cancer from stromal cells, and (2) while the irradiated feeder fibroblasts enhanced the growth rate of cancer cells (for example, MGH121-1, MGH134-1, MGH707-1, MGH045-1, and MGH051-1), resistance to EGFR or ALK inhibition is not conferred by the irradiated fibroblast feeder layer.

Since TCM tended to help the growth of biopsy cultures, we wanted to determine whether this assay could be performed on cells growing in TCM. Therefore, we tested the ability of the growth media to influence drug response. We first grew established *EGFR* mutant and *ALK*-translocated NSCLC cell lines in either TCM or R10 media and treated them with EGFR or ALK inhibitors, respectively (Figure S2). Cells grown in TCM media (Figure S2, blue curves) displayed a rightward shift in half maximal inhibitory concentration (IC_50_) compared to cells grown in R10 media (red curves). However, when epidermal growth factor (EGF) and insulin were removed from TCM media (TCM, -E, -I media; Figure S2, orange curves), the drug response more closely mimicked the response in R10 media (Figure S2, red curves). These studies suggest that exogenous EGF and insulin could reduce the sensitivity of *EGFR* mutant and *ALK*-translocated NSCLC cells to EGFR or ALK inhibitors, respectively, and should be removed from the media prior to drug sensitivity assays. We also compared the response of the established *EGFR* mutant and *ALK*-translocated patient-derived NSCLC cell lines to EGFR or ALK inhibition growing in the presence of TCM media without EGF and insulin (TCM, -E, -I media; Figures 2I–2K, orange curves) to that of the same cells grown in their native media, R10 (Figures 2I–2K, blue curves), in the presence of irradiated feeder fibroblasts (i.e., mixed cultures). Despite a significant growth advantage for certain cancer cells in TCM, -E, -I media (MGH121-1 and MGH045-1), their response to EGFR or ALK inhibition was nearly identical to cells growing in R10 media. These data suggest that other factors in the TCM, -E, -I media, including the ROCK inhibitor, do not change the response of *EGFR* mutant or *ALK*-translocated lung cancer cells to EGFR or ALK inhibition compared to R10 media. Taken together, these data not only show the consistency of the immunofluorescence assay compared to a standard measure of cell viability, but they also suggest that assay conditions (feeder fibroblasts and TCM, -E, -I media) could be identified that do not alter the response of *EGFR* mutant or *ALK*-rearranged lung cancer cells to EGFR or ALK tyrosine kinase inhibitors (TKIs), respectively.

### The Response of NSCLC Patients’ Biopsy Cultures Correlates with Patients’ Responses

We utilized the high-throughput immunofluorescence assay to test the ability of patient-derived NSCLC cultures to predict patients’ responses to targeted therapies. We established cell culture models from biopsies or pleural effusions of NSCLC patients whose disease progressed on treatment with a first- and/or second-generation EGFR or ALK TKI and responded to a second- or third-generation inhibitor. The patients’ treatment schedules prior to biopsy, biopsy genetics, subsequent therapy, and RECIST (Response Evaluation Criteria In Solid Tumors) response to that therapy are provided in Table 2. The workflow, for what we termed the functional assessment of tumors (FAsT), is detailed in Figure S3. Twenty-four hours prior to plating the patient-derived cell culture, a monolayer of irradiated feeder fibroblasts was established in 384-well plates. Patient-derived cell cultures were plated at roughly 250–1,000 cancer cells per well into 384-well plates that included a plate to fix on the day of treatment initiation (day-0 plate), a drug plate, and growth plates. The drug plate consisted of a 12-dose treatment of the same or a similar (same generation of TKI with a similar mutated target-targeting capacity) therapy for which the patient was resistant and subsequently responsive, as well as a therapy that should be innocuous—for example, an ALK inhibitor in an *EGFR* mutant culture. Treatments were done in quadruplicate. Growth plates allowed us to monitor the growth rate of the cancer (CK8/18-positive) and non-cancer (CK8/18-negative) cells in the culture and, therefore, determine when to stop the assay and process the drug plate for viability assessment. We chose to allow for approximately two cancer cell doublings. After drug plate fixation, immunofluorescence was performed with the primary rabbit anti-CK8/18 antibody cocktail, secondary goat anti-rabbit IgG (immunoglobulin G)-Alexa Fluor 684 antibody and Hoechst 33342. 384-well plates were imaged using Molecular Devices’ ImageXpress Micro XL high content imager, and cell scoring was accomplished with their MetaXpress software.

**Table 2:** Clinical Information of *EGFR-*Mutant and *ALK-*Translocated Early Biopsy Cultures

MGH ID	Oncogenic Driver	Targeted Therapy (or Therapies) Preceding Biopsy	Biopsy Site	Biopsy Genetics	Subsequent Therapy	Best RECIST Response	TTP (Months)
MGH707-1	EGFR exon 19 del	erlotinib	lung	T790M	rocelitinib	PR −49.9%	ND
		afatinib+cetuximab					
MGH721-1	EGFR exon 19 del	erlotinib	lung	T790M	rocelitinib	PR −51.7%	12.1
MGH748-1	EGFR exon 19 del	gefitinib	lymph node	T790M	osimertinib	PR −72.1%	14.6
		erlotinib					
		afatinib					
		icotinib					
		afatinib+cetuximab					
MGH832-1	EGFR exon 19 del	afatinib	lung	WT MET amplification (>25:1)	erlotinib+crizotinib	ND	ND
MGH021-2	ALK fusion	crizotinib	pleural effusion	G1202R, G1269A, 1151Tins, I1322M	ceritinib	PR −51.8%	2.8
MGH051-1	ALK fusion	crizotinib	liver	WT	ceritinib	PR −53.0%	7.5
MGH092-1	ALK fusion	crizotinib	lung	G1202 deletion	lorlatinib	PR −47.5%	4.4
		ceritinib					
		crizotinib					

ND, not determined; PR, partial response; WT, wild-type.

**Figure 3:**
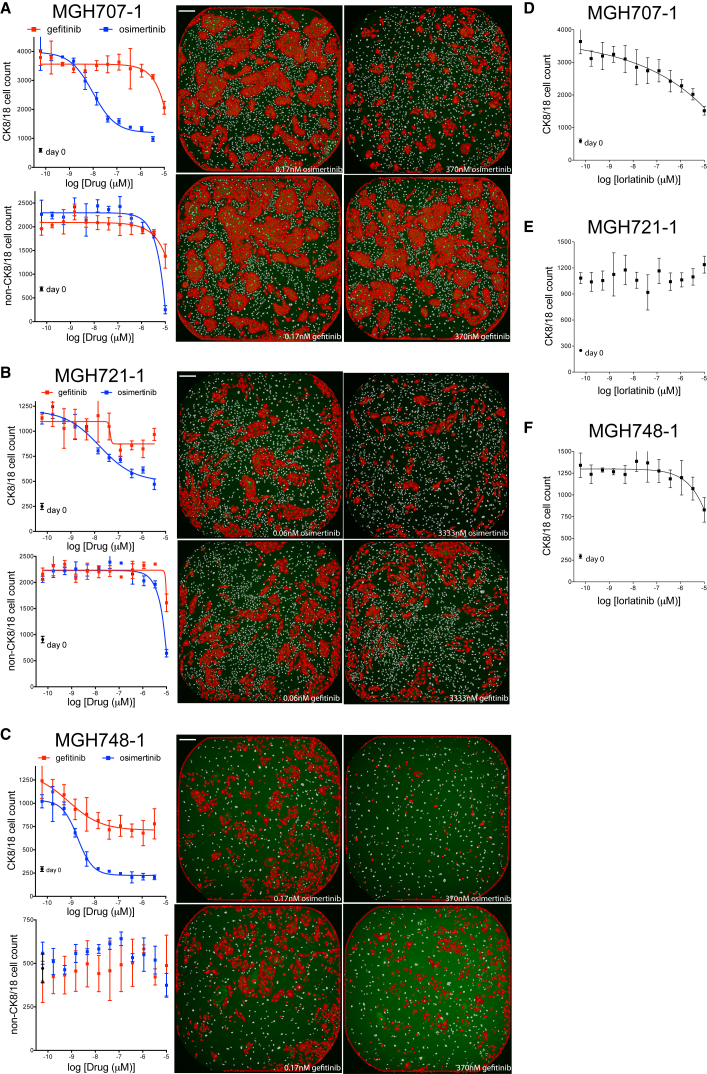
Response of *EGFR-*Mutant Biopsy Cultures MGH707-1, MGH721-1, and MGH748-1 to EGFR inhibitors or an ALK Inhibitor (A–F) The early-biopsy cultures of *EGFR*-mutant lung cancers MGH707-1 (A and D), MGH721-1 (B and E), and MGH748-1 (C and F) were plated on a monolayer of ∼500 irradiated feeder fibroblasts in TCM, -E, -I media in 384-well plates and treated with 12 doses of the EGFR inhibitors gefitinib or osimertinib (A–C) or the ALK inhibitor lorlatinib (D–F) for 6 days. Plates were fixed and stained with Hoechst 33342 and the anti-CK8/18 antibody to determine the change in CK8/18-positive and CK8/18-negative cells. CK8/18-positive cell counts are illustrated in the top graph, and CK8/18-negative cell counts are depicted on the bottom. Circles indicate cell number at day of treatment initiation (day 0). Nonlinear regression curves fit to the data points are shown, and data are represented as mean ± SD with n = 4 replicate wells. Representative images of low and high doses of gefitinib or osimertinib are shown on the right. Scale bars, 270 μm.

The *EGFR* mutant NSCLC patients from whom models MGH707-1, MGH721-1, and MGH748-1 were derived progressed on first- and/or second-generation EGFR inhibitor regimens, including erlotinib, gefitinib, icotinib, and afatinib, alone and in combination with the anti-EGFR antibody cetuximab (Table 2). Genetic analysis of the patients’ biopsies revealed a T790M mutation in EGFR, an acquired resistance mechanism (Kobayashi et al., 2005, Pao et al., 2005); therefore, the patients were prescribed a third-generation inhibitor, either rociletinib or osimertinib, known to inhibit the gatekeeper residue mutation EGFR T790M. These patients exhibited a partial response to the third-generation inhibitor. The core biopsies of these patients were cultured in TCM media on a layer of irradiated feeder fibroblasts. Once the cultures reached approximately >70% confluence in a 6-cm dish at 17, 15, and 9 weeks, a portion of cells was viably frozen before a pure cancer cell culture was established (Table S8). We thawed these viably frozen biopsy cultures on feeder+TCM and tested their response to EGFR inhibition. At the time of cell plating, the biopsy cultures contained 5.1, 3.4, and 2 million total cells, respectively, corresponding to roughly 3, 1, and 2 million cancer cells, as depicted by the ratio of CK8/18-positive to CK8/18-negative (non-feeder) cells on the day-0 plates (Table S8). We transferred the cells to 384-well plates in TCM, -E, -I media coated with ∼500 irradiated feeder fibroblasts as described earlier. Between 250 and 500 CK8/18-positive (cancer) cells were plated per well, and in some cultures, CK8/18-negative (non-CK8/18) cells, other than feeder fibroblasts, were observed. For consistency, the drug plates for these biopsy cultures consisted of the first-generation EGFR inhibitor gefitinib and the third-generation EGFR inhibitor osimertinib. Drug treatment was performed in quadruplicate for each dose. The growth of each culture was monitored via growth plates that were serially fixed and scored in parallel, and the drug plates were fixed when the cancer cell population (CK8/18 positive) had, at least, quadrupled. Representative images are shown at the right of each graph in Figure 3.

As illustrated in Figure 3, a significant number of non-CK8/18 cells were present in the biopsy cultures of MGH707-1 (Figure 3A) and MGH721-1 (Figure 3B). The morphology of these cells suggested a fibroblast cell, and these cells grew as well as or better than the CK8/18-positive cells over the course of the experiment. These CK8/18-negative cells were unresponsive to gefitinib or osimertinib (Figures 3A and 3B, bottom graphs). The basal level of non-CK8/18 cells in the MGH748-1 culture (Figure 3C) is likely a quantification of only irradiated fibroblast feeder cells (and not tumor-derived stromal cells), since 500 irradiated feeder fibroblasts were plated (consistent with day 0), and this number did not increase over time. As illustrated in Figures 3A and 3B, as few as 250 cancer cells per well of a 384-well plate at the day of treatment initiation (day 0) were sufficient to determine a significant difference between low-dose and high-dose treatment, at least after two cell doublings. The osimertinib IC_50_ for CK8/18-positive cells in MGH707-1, MGH721-1, and MGH748-1 biopsy cultures were in the single- or double-digit nanomolar concentration, consistent with high sensitivity on target and the ability of the third-generation inhibitor to overcome T790M mutation of EGFR (Cross et al., 2014). Erlotinib had minimal efficacy on CK8/18-positive cells in MGH707-1 and MGH721-1 biopsy cultures and intermediate efficacy on MGH748-1 at concentrations as high as 3.33 μM. Furthermore, the ALK inhibitor lorlatinib failed to suppress the growth of these *EGFR* mutant biopsy cultures (Figures 3D–3F).

**Figure 4:**
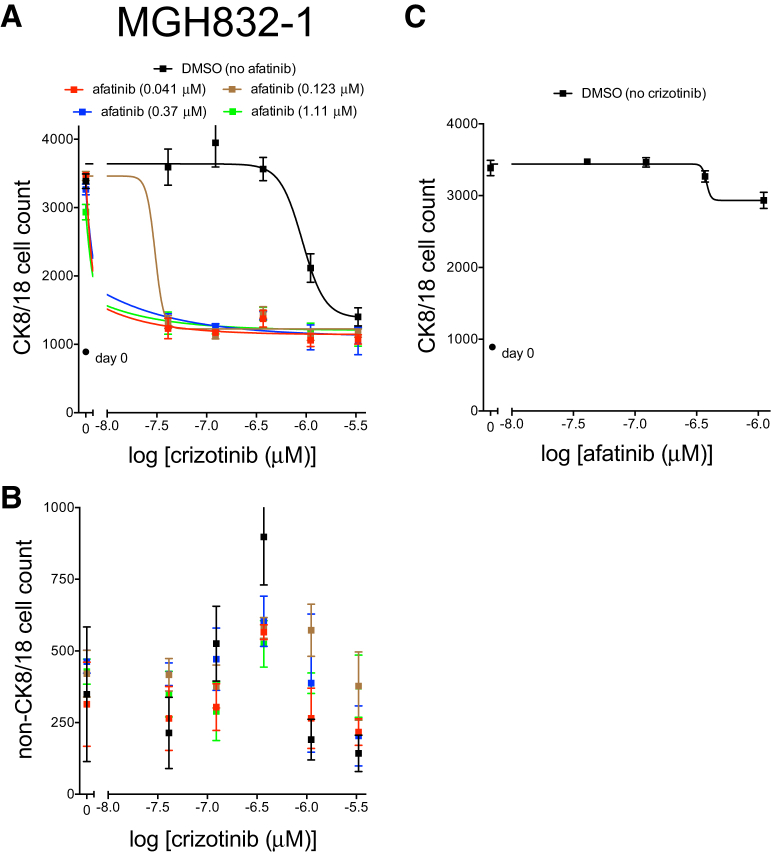
Response of Biopsy Culture MGH832-1 (*EGFR* Mutant, *MET* Amplified) to Afatinib and/or Crizotinib (A–C) The early-biopsy culture of MGH832-1 was plated on a monolayer of ∼500 irradiated feeder fibroblasts in TCM, -E, -I media in 384-well plates and treated with the indicated doses of the EGFR inhibitor afatinib (C) and/or the MET inhibitor crizotinib (A and B) for 6 days. Plates were fixed and stained with Hoechst 33342 and the anti-CK8/18 antibody to determine the change in CK8/18-positive cells (A and C) and CK8/18-negative cells (B). Circles indicate cell number at day of treatment initiation (day 0). Nonlinear regression curves fit to the data points are shown, and data are represented as mean ± SD with n = 4 replicate wells.

The *EGFR* mutant NSCLC model MGH832-1 was derived from a patient who failed afatinib therapy (Table 2). The identification of MET amplification in the patient’s tumor, a well-established mechanism of resistance to anti-EGFR therapy (Engelman et al., 2007), led to the prescription of a combination of erlotinib and the MET inhibitor crizotinib. The patient did not tolerate the combination due to toxicity and discontinued crizotinib at approximately 1 month of treatment. No restaging scans were performed. The core biopsy culture reached approximately >70% confluence in a 6-cm dish, resulting in 4 million cells in 8 weeks (Table S8). The culture was plated onto a feeder fibroblast layer in 384-well plates at 1,000 cells per well and treated with afatinib alone or with increasing concentrations of the MET/ALK inhibitor crizotinib. As depicted in Figure 4, afatinib monotherapy had a minimal effect on CK8/18-positive cell number as high as 1 μM. However, the combination of afatinib and crizotinib, at concentrations as low as 41 nM each, resulted in significant suppression of the CK8/18-positive cell number (Figure 4). The functional data are consistent with the genetics of this patient’s tumor and previous clinical data with this combination (Gainor et al., 2016b, Scheffler et al., 2015).

**Figure 5:**
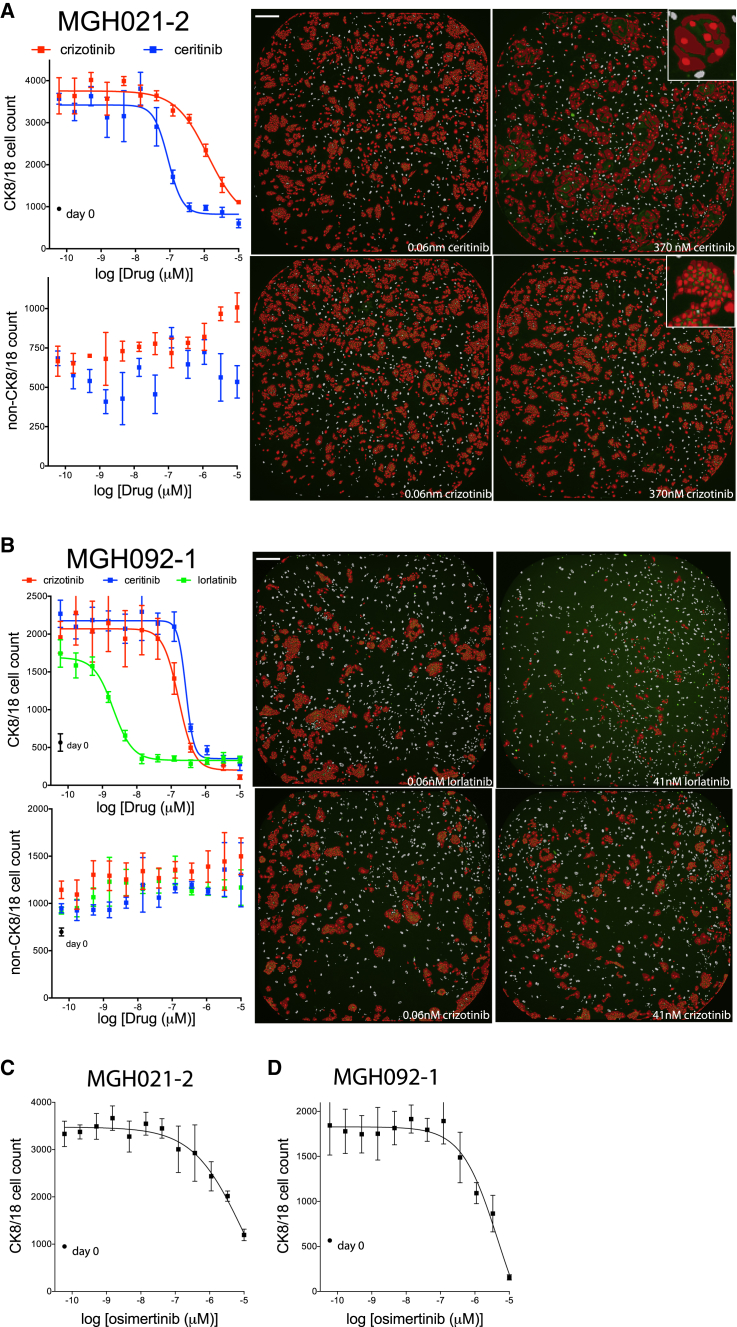
Response of *ALK-*Translocated Biopsy Cultures MGH021-2 and MGH092-1 to ALK Inhibitors or an EGFR Inhibitor (A–D) The early-biopsy cultures of *ALK*-translocated lung cancers MGH021-2 (A and C) and MGH092-1 (B and D) were plated on a monolayer of ∼500 irradiated feeder fibroblasts in TCM, -E, -I media in 384-well plates and treated with 12 doses of the indicated ALK inhibitors (A and B) or the EGFR inhibitor osimertinib (C and D) for 6 days. Plates were fixed and stained with Hoechst 33342 and the anti-CK8/18 antibody to determine the change in CK8/18-positive and CK8/18-negative cells. CK8/18-positive cell counts are illustrated on the top graph and CK8/18-negative cell counts are depicted on the bottom. Circles indicate cell number at day of treatment initiation (day 0). Nonlinear regression curves fit to the data points are shown, and data are represented as mean ± SD with n = 4 replicate wells. Representative images of low and high doses of crizotinib or ceritinib are shown on the right. Scale bars, 270 μm.

The ALK-translocated NSCLC patients’ models MGH021-2 and MGH051-1 were derived following progression on the first-generation ALK inhibitor crizotinib (Table 2). Multiple secondary alterations in the ALK kinase domain were observed in MGH021-2, while no mutations were detected in the ALK kinase domain of MGH051-1. The second-generation ALK inhibitor ceritinib has been shown to overcome crizotinib resistance in patients with particular ALK kinase mutations as well as in patients without detectable mutations (Friboulet et al., 2014, Gainor et al., 2016a, Shaw et al., 2014). Consistent with these findings, both patients had a partial response to ceritinib. The ALK-translocated NSCLC model MGH092-1 was derived following progression on prior crizotinib and ceritinib. A G1202 deletion was detected in the ALK kinase domain, which has been shown to mediate ceritinib resistance but remains sensitive to lorlatinib (Gainor et al., 2016a). These three cultures reached approximately >70% confluence in a 6-cm dish at 13, 20, and 16 weeks from biopsy (Table S8). This corresponded to 4, 3, and 1.5 million total cells—roughly all cancer cells, as depicted by the lack of CK8/18-negative non-feeder cells in the day-0 culture plates. The pleural effusion of MGH021-2 and the core biopsies of MGH051-1 and MGH092-1 were cultured in TCM, -E, -I media on a layer of irradiated feeder fibroblasts, and their responses to ALK inhibitors were tested, similarly to the method described earlier. Representative images are shown at the right of each graph in Figures 5A, 5B, and S4A.

As illustrated in Figure 5A, the ceritinib IC_50_ for CK8/18-positive cells in the MGH021-2 culture was approximately 90 nM, greater than 14-fold lower than the IC_50_ for crizotinib (∼1,300 nM). Note that, while low-magnification (4×) microscopic images did not show a dramatic response, closer examination illustrated a change in cell morphology under ceritinib treatment (Figure 5A, insets). The cancer cells treated with 370 nM ceritinib were bigger and, therefore, occupied much of the well; however, there were much fewer CK8/18-positive nuclei within the high-dose treatment well. In addition to distinguishing between cell types, microscopy has the added benefit of the observance of cell-morphological changes. The ceritinib and crizotinib IC_50_s for CK8/18-positive cells in the MGH051-1 culture (Figure S4) were nearly identical (53 and 60 nM, respectively), consistent with the crizotinib IC_50_ for the finished, pure cancer cell line that we previously reported (Friboulet et al., 2014). Consistent with these data, ceritinib has demonstrated clinical activity in crizotinib-resistant *ALK*-positive tumors without *ALK* mutation or gene amplification (Shaw et al., 2014). Figure 5B displays the data with the biopsy culture of patient sample MGH092-1 (G1202 del). The lorlatinib IC_50_ for CK8/18-positive cells in the MGH092-1 culture was in the single-digit nanomolar concentration (∼2 nM), at least 83-fold greater than the IC_50_s for crizotinib or ceritinib (166 and 268 nM, respectively), consistent with the unique activity of lorlatinib against this specific mutation (Gainor et al., 2016a). Finally, the EGFR inhibitors osimertinib or rociletinib failed to suppress the growth of these *ALK*-translocated biopsy cultures (Figures 5C and 5D).

## Discussion

Technological advances in DNA, RNA, and protein analyses over the past decade have provided hope of personalized care for cancer patients. However, an incomplete understanding of the relationship between tumor genotype, RNA and protein expression, and tumor phenotype limits the utility of these technologies for personalized care. A functional test of a patient’s cancer cells may overcome this limitation and is currently being clinically tested in hematological malignancies (clinicaltrials.gov identifier NCT: NCT01620216). The challenge for culturing cancer cells from solid tumors, however, is greater. Organoid cultures have been used to culture patients’ cancer cells, and while the success rate is reasonable, it is not clear whether the turnaround time is quick enough to impact patient care (Pauli et al., 2017).

One major obstacle we experienced in culturing lung cancers has been the minimal number of cancer cells that can be obtained from a core biopsy, particularly one designated for research purposes. Our data suggest that starting material is a critical variable and that greater success will result from tissue samples with more cancer cellularity. Note that, in these low cancer cellularity cores, we observed either very little viable cells or only fibroblasts but did not identify normal epithelial cells based on visual assessment. Since pleural effusions likely harbor higher cancer cellularity and do not require the feeder+TCM culture condition, pleural effusions offer an immediate path forward to perform a functional test within a month of sample collection.

In our hands, the feeder+TCM culture condition tended to be the most successful in developing a cancer culture from a patient’s tumor sample. While exciting, the feeder+TCM culture conditions provide challenges for cancer cell sensitivity assessment. Liu et al. recently demonstrated the robust culture of normal epithelium using a similar culture system (Liu et al., 2017). While we rarely observe normal epithelial cells when growing cultures of CT (computed tomography)-guided biopsies of metastatic lesions, next-generation DNA sequencing can be applied to confirm the allelic fraction of oncogenic mutations. An alternative option to discriminate between tumor and normal epithelium is the use of a cancer-cell-specific antibody. For example, we found marked consistency of ALK and CK8/18 staining for *ALK*-translocated NSCLC cells.

Another challenge with the feeder+TCM culture condition is the presence of growth factors. The EGF and insulin in TCM media shifted the IC_50_ curves for *ALK*-translocated and *EGFR* mutant NSCLC cells. Therefore, cancer cells of early-biopsy cultures must be weaned off of EGF and insulin prior to drug testing. This could be a potential hurdle for cancer cell expansion to obtain sufficient number in an acceptable time frame using early-biopsy NSCLC cells. Additionally, adapting the approach to other cancer types and/or other therapeutics will require thorough testing of the culture conditions to avoid artificial *in vitro* effects.

The immunofluorescence-based assay described in this article overcomes a number of obstacles previously hindering the functional examination of patients’ tumors. The assay was capable of determining response in as few as 100 cells per well, overcoming the problem of minimal viable cancer cells in a core biopsy and the quick turnaround time necessary to impact patient care. In our experience with early cultures of patients’ tumor samples, the acquisition of millions of cells after <3 months of culture is feasible. We analyzed 61 lung cancer samples for the time it took to reach approximately >70% confluence in a 6-cm dish confluence (Table S9). For all samples, we found that the average time was 11.5 weeks. Of 61 lung samples with detailed timing information, 17 reached target confluence in less than 31 days (mostly pleural effusions). To put this time frame in perspective, the standard turnaround time for clinical genomic tests is 2 weeks, but this type of assay has been under development, leveraging large resources across cancer centers for over 10 years. Two million cells would allow for the testing of roughly 100 drugs in a 10-point dose-response curve per drug if each dose was done in quadruplicate. Obviously, if fewer drugs were tested, the assay could be run earlier.

The ability to test cancer cell drug sensitivity in cultures with feeder fibroblasts and tumor stromal cells overcomes the time it takes to develop a culture devoid of stroma. In addition to speeding up the time from biopsy to drug testing, this ability allows for a potentially better representation of tumor response—due to the presence of tumor stromal cells—and increased sample success, as establishing a pure cancer cell line provides greater challenges. Finally, the data suggest that the culture conditions of the assay, including media components and feeder fibroblasts, do not induce response differences from standard culture media and clinical response expectations.

While we have found consistent responses of early-biopsy cultures with patients’ responses for TKIs, we do not imply that the same will hold true for drugs of different mechanisms of action. We believe that this question represents an important aspect of model establishment for functional diagnostic across disease subtypes and needs to be addressed prospectively.

Based on these encouraging results and our ability to test clinical drug effects on complex cultures derived from biopsies using a relatively simple assay, we believe that a co-clinical trial testing the diagnostic value of this approach in complement to genotyping is feasible. Our findings suggest that the functional test can identify combination partners within a genetically defined population, but the question remains as to whether it can do so in an undefined one. A co-clinical exploration of combination therapies to overcome EGFR and ALK therapeutic resistance may provide an opportunity to test the robustness of this approach.

## Experimental Procedures

### Tumor Sample Processing

All human lung cancer samples were obtained from patients, with their informed consent, at the Massachusetts General Hospital (MGH), and all procedures were conducted under an Institutional Review Board (IRB)-approved protocol. Patients were all adults; age and sex were not considered as relevant factors in these studies. Patient biopsies and resections were placed in a sterile conical tube containing ACL4 media (Invitrogen) with 10% FBS and 1% antibiotic-antimycotic (Fisher Scientific) on wet ice during transport from the operating room to the research laboratory. Pleural effusions were sent on wet ice. A courier service allowed for the arrival of tumor tissue at the laboratory within 30 min of clinical sample collection. Upon arrival, biopsies and resections were manually minced using a sterile scalpel. The majority of patient biopsies underwent an enzymatic digestion with 25 μg/mL liberase (in 5 mL ACL4 media) for 1 hr in a 37°C MultiTherm shaker (Benchmark Scientific) set at 1,000 rpm. Resections were digested using Miltenyi Biotec’s Tumor Dissociation Kit and gentleMACs Dissociator with heaters. Dissociation was stopped by adding 0.5 mL FBS. Pleural effusions underwent a 10-min centrifugation at 440 × *g*, followed by a 10-min red blood cell lysis using the appropriate buffer (BioLegend). After digestion or lysis, cells were separated into a number of plating conditions, dependent on tissue pellet size. These included collagen-coated dishes with RPMI and 10% FBS (R10), DMEM and 10% FBS (D10), or ACL4 and FBS (A < 5 and A ≥ 5) media, as well as dishes coated with irradiated human foreskin fibroblasts and TCM (feeder+TCM; discussed later).

Primary human foreskin fibroblasts were kindly provided by Dr. William Michaud at MGH. Adherent foreskin fibroblasts were irradiated at 5,000 rads in 15-cm dishes. After at least 24 hr, irradiated fibroblasts were seeded in 6-cm dishes at a density of 140,000 cells. After adherence, the irradiated-fibroblast plates were used for sample plating. TCM contained the following reagents: 375 mL F/12+GlutaMAX, 125 mL DMEM with L-glutamine, 4.5 g/L glucose without sodium pyruvate, 5% FBS, 1% antibiotic-antimycotic, 4 μg/mL hydrocortisone (Sigma); 1 μg/mL insulin (Sigma); 10 ng/mL EGF (PeproTech); 1.68 ng/mL cholera toxin (Sigma); 12 mg adenine (Sigma); and 10 M ROCK inhibitor Y-27632 (Selleckchem).

Patient-derived tumor cell cultures were passaged until a tumor majority was observed. At that time, a portion was viably frozen. These early biopsy cultures were potentially of mixed-cell populations and were utilized in Figures 3, 4, 5, S1, and S4. To develop established/finished cancer cell lines, these early cultures were passaged off of the feeder and processed to rid of stromal cells. A cell line was determined “finished” when the culture was independent of the fibroblast feeders, free of stromal cells, and determined to maintain known patient tumor mutations.

### Immunofluorescence Assay

We developed an immunofluorescence assay that allowed for the identification of epithelial cells using a cocktail of antibodies against cytokeratin 8 and cytokeratin 18. Cell cultures were first fixed with 3.75% formaldehyde for 30 min at room temperature (RT) and washed three times with PBS. The BioTek ELx405 microplate washer was used for all washing steps. The primary antibody CK8/18 cocktail (clone EP17/30, Dako, M3652) was diluted 1:100 in PBS with 1% goat serum and 0.1% Triton X-100 and incubated for 20–24 hr at 4°C. The wells were washed three times with PBS before the addition of an Alexa Fluor 647-tagged goat anti-rabbit secondary antibody (Life Technologies, A21245) at a 1:100 dilution in 1% goat serum and 0.1% Triton X-100. After overnight incubation at 4°C with the secondary antibody in the dark, Hoechst 33342 was added to the well at a final concentration of 4 μg/mL and incubated at RT for 2 hr in the dark. Finally, the wells were washed three times with PBS and stored in ∼50 μL of PBS with an aluminum cover to prevent room light excitation.

### High-Content Imaging and Image Analysis

Imaging of the immunofluorescence-stained cultures was performed with Molecular Devices’ ImageXpress Micro XL high-content imager. Briefly, the post-laser z-offset was determined for correct autofocusing, and the exposure time for each illumination filter was calculated. Several wells across the 384-well plate were tested for consistency prior to acquisition of the entire plate.

Analysis of the fluorescent images was done with Molecular Devices’ MetaXpress software and their Multi-Wavelength Cell Scoring Application Module. Briefly, the minimum and maximum widths as well as the signal intensity above local background were determined for proper segmentation of the nuclear Hoechst 33342 stain and the cytoplasmic CK8/18 stain (entire cell). Several wells of the 384-well plate were previewed by eye for accurate segmentation prior to analysis of the entire plate. Data collected from the analysis included the number of total cells (Hoechst 33342-positive nuclei count), the number of epithelial cells (Hoechst 33342-positive and CK8/18-positive cell count), and the number of non-epithelial cells (Hoechst 33342-positive and CK8/18-negative cell count).

### Compounds

Gefitinib, osimertinib, rocelitinib, crizotinib, ceritinib, and lorlatinib were obtained from Selleck Chemicals.

### Statistical Analyses

Analyses of the cancer cell culture success rates between tumor type, tissue type, biopsy site, and media type were done using the Fisher’s exact test. All non-linear curve fit analyses were done with GraphPad Prism software.

## Supplemental Information

Document S1. Figures S1–S4 and Tables S1–S9

Document S2. Article plus Supplemental Information
