# Supplementary material for: Primary Patient-Derived Cancer Cells and Their Potential for Personalized Cancer Patient Care
Source: Cell Rep. 2017 Dec 12;21(11):3298–309. doi: 10.1016/j.celrep.2017.11.051 (PMC5745232; doi:10.1016/j.celrep.2017.11.051)
Supplement: Document S1. Figures S1–S4 and Tables S1–S9 [file mmc1.pdf]

Cell Reports, Volume *21*

Supplemental Information

Primary Patient-Derived Cancer Cells and Their Potential for Personalized Cancer Patient Care

David P. Kodack, Anna F. Farago, Anahita Dastur, Matthew A. Held, Leila Dardaei, Luc Friboulet, Friedrich von Flotow, Leah J. Damon, Dana Lee, Melissa Parks, Richard Dicecca, Max Greenberg, Krystina E. Kattermann, Amanda K. Riley, Florian J. Fintelmann, Coleen Rizzo, Zofia Piotrowska, Alice T. Shaw, Justin F. Gainor, Lecia V. Sequist, Matthew J. Niederst, Jeffrey A. Engelman, and Cyril H. Benes

Supplemental Information

Supplemental Tables

Table S1. Success of Breast Cancer Cell Line Generation by Subtype. Related to Table 1.

Breast Cancer Subtype	Number Finished	Number Failed	Total Processed	Percent Successful
Basal (ER-, PR-, HER2-)	2	7	9	22
Luminal	11	72	83	13

Table S2. Subjective Reasons for the Failure of Cancer Cell Line Generation. Related to Table 1.

Failure Reason	Number of Failures	Percent of Failures
No/few cancer cells identified after dissociation	221	77
Stroma outgrowth	41	14
Cancer cells died/didn't proliferate	24	8

Table S3. Success of Lung Cancer Cell Line Generation by Tissue Type. Related to Table 1.

Tissue Type	Number Finished	Number Failed	Percent Successful
Core Biopsy/ FNA	43	141	23
Pleural Effusion	56	76	42
Resection	5	9	36
Autopsy	3	20	13

Table S4. Success of Lung Cancer Cell Line Generation by Biopsy Site. Related to Table 1.

Biopsy Site	Number Finished	Number Failed	Percent Successful
Lung	16	48	25
Liver	15	27	36
Lymph Node	2	12	14
Bone	1	1	50

Table S5. Analysis of Feeder+TCM Culture Compared to All Other Culture Conditions (R10, D10, ACL4<5 or ACL4≥5) within Each Tissue Type for All Finished Lung Cancer Cell Lines Grown in Multiple Conditions. Related to Table 1. Data shown as a percent and total number in parentheses.

Tissue Type	Percent Success of Feeder+TCM (number)	Percent Success of Any Other Media (number)	Percent Success of Both (number)	Total
Core Biopsy/ FNA	46 (12)	23 (6)	31 (8)	26
Pleural Effusion	35 (9)	19 (5)	46 (12)	26
Resection/Autopsy	22 (2)	33 (3)	44 (4)	9
Total	37 (23)	22 (14)	41 (24)	61

Table S6. Comparison of the Ability of the Indicated Antibodies to Identify Epithelial Cancer Cells. Related to Figures 1-2. CK, Cytokeratin; EPCAM, Epithelial cell adhesion molecule; * denotes cells that have undergone epithelial-to-mesenchymal transition; -, no staining; +/-, minimal staining; +, low staining; ++, moderate staining; +++, strong staining

Cell line Identifier	Cancer Type	Pan-CK clones AE1/AE3	Pan-CK clone MNF116	CK5/6	CK7	CK8/18	CK20	EPCAM	Pan-CK clone CAM5.2	E-cadherin
Human foreskin fibroblasts	-	-	-	-	-	-	+/-	-	+/-	
PC-9	lung	++	+++	-	+++	+++	-	+/-	++	+/-
H1650	lung	+	++	-	+++	+++	-	+/-	++	+/-
A549	lung	+	++	-	++	+++	-	+	+	+/-
H1975	lung	++	+	-	+++	++	-	-		+
H1975-R2*	lung	+	+/-	-	++	+	-	-		+/-
MGH048	lung	+/-	++	-	+/-	+++	-	+/-	+/-	-
MGH065*	lung	-	-	-	-	++	-	-		-
MGH068	lung	++	+		+	+++			+/-	
MGH084	lung	+++	+/-	-	++	+++	-			
MGH092	lung	++	++	-	+++	+++	-	++		
MGH121	lung	+	++	-	++	+++	-	+/-	+	+/-
MGH134	lung	+	++	-	++	+++	-	+/-	+	+/-
MGH157	lung	+	++	-	++	+++	-	+/-	+	+/-
MGH174	lung	+	+	-	+	++	-	+/-		
MGH505	lung	+/-	+/-		+/-	+/-	-			
MGH707	lung	+	+/-	-	++	+++	-	+/-		
MGH712	lung	+/-	++	-	+++	+++	-	+		
MGH722	lung	+	++	-	+++	+++	-	+/-		
MGH732	lung	+/-	+/-	-	++	++	-	+/-		
MGH748	lung	+/-	+	-	-	+++	-	+/-	+/-	-
MGH757	lung	+/-	+/-	-	++	+	-			
MGH778	lung	+/-	+/-	-	+/-	+	-			
MGH781	lung	+	+/-	-	+/-	++	-			
MGH785	lung	+/-	+	-	+	+	-			
MGH6007	lung	+/-	+/-	-	+/-	+	-			
MGH6009	lung	+	+/-	-	++	+++	-	-		
MGH902	lung	+/-	+/-		+/-	++			+/-	
MGH334	breast	+/-	+/-	-	+/-	+++	-			
MGH421	breast	+/-	++	-	++	+++	-	+		
MGH345	bladder	+	++	-	+/-	++	-	+/-		
MGH620	colorectal	+/-	+	-	-	++				
MGH616	colorectal	++	+/-	-	-	+++	+/-	+++		
MGH603	colorectal	-	-	-	-	++	+/-	++		
MGH211	colorectal	+/-	-	-	-	+++	+	+++		
MGH439	gallbladder	+/-	+/-	-	+/-	++	-			

Table S7. Ability of the CK8/18 Antibody Cocktail to Stain Cancer Cells. Related to Figures 1-2. +, low staining; ++, moderate staining; +++, strong staining

Cell line Identifier	Cancer Type	CK8/18 score
1A6	bladder	++
HT-1197	bladder	++
T24	bladder	+++
UACC893	breast	+++
BT483	breast	+++
HDQ-P1	breast	+++
COLO-206F	intestine	+++
COLO-320	intestine	++
C170	intestine	++
H2170	lung, squamous	++
H520	lung, squamous	++
SW900	lung, squamous	++
H1694	SCLC	+
H1092	SCLC	+++
H2061	SCLC	++
HT-29	CRC	+++
HCT116	CRC	+++
COLO-326	CRC	++
AsPC-1	pancreas	++
BxPC-3	pancreas	++
PANC-1	pancreas	+++
SK-MEL-3	melanoma	+
SK-MEL-28	melanoma	+
WM164	melanoma	+

Table S8. Culture information of *EGFR*-mutant and *ALK*-translocated early biopsy cultures. Related to Figures 3-5. Time to viable freeze of 6-cm culture dish when approximately >70% confluent (in weeks), and the approximate cancer cell number and total cell number at time of the assay are given.

MGH ID	Time to Freeze (weeks)	Approximate Cancer Cell Number and Total Cell Number (millions)
MGH707-1	17	3 (5.1)
MGH721-1	15	1 (3.4)
MGH748-1	9	2 (2)
MGH832-1	8	4 (4)
MGH021-2	13	4 (4)
MGH051-1	20	3 (3)
MGH092-1	16	1 (1)

Table S9. Primary culture time frame. Related to Figures 3-5. Time to viable freeze of 6-cm culture dish when approximately >70% confluent (in days) and sample type for each primary cell model are shown. * denotes multiple samples from the same patient.

MGH ID	Sample Type	Time to Freeze (days)
MGH023-2	Biopsy	58
MGH034-2	Biopsy	150
MGH044-1	Biopsy	251
MGH045-1*	Resection	311
MGH045-2*	Fluid	27
MGH048-1*	Fluid	68
MGH048-4*	Fluid	14
MGH048-5*	Fluid	57
MGH049-1	Fluid	96
MGH051-2	Biopsy	31
MGH056-1	Biopsy	112
MGH064-1	Fluid	97
MGH065-1*	Biopsy	36
MGH065-3*	Biopsy	13
MGH068-2	Biopsy	19
MGH073-1*	Fluid	21
MGH075-2*	Fluid	71
MGH075-3*	Fluid	68
MGH083-3	Fluid	28
MGH084-1	Biopsy	90
MGH085-1	Fluid	111
MGH092-1	Biopsy	113
MGH1088-1	Fluid	80
MGH119-2	Biopsy	50
MGH121-2	Fluid	166
MGH143-3	Biopsy	55
MGH144-1	Fluid	28
MGH148-3	Fluid	81
MGH164-1*	Fluid	128
MGH164-2*	Fluid	21
MGH164-3*	Resection	28
MGH177-1	Fluid	22
MGH180-1	Biopsy	41
MGH212-1	Fluid	27
MGH306-1	Biopsy	43
MGH308-1	Biopsy	99
MGH503-1	Fluid	37
MGH505-1	Fluid	56

MGH700-2	Biopsy	42
MGH706-2	Biopsy	47
MGH707-1	Biopsy	119
MGH707-2	Biopsy	50
MGH709-1	Biopsy	226
MGH709-2	Biopsy	67
MGH712-1	Resection	141
MGH721-1	Biopsy	104
MGH725-1	Biopsy	204
MGH741-1	Fluid	77
MGH744-1	Fluid	57
MGH748-1	Biopsy	64
MGH754-2	Biopsy	22
MGH800-1	Biopsy	13
MGH802-2	Biopsy	42
MGH805-1	Biopsy	163
MGH809-1	Biopsy	42
MGH810-1	Fluid	7
MGH814-1	Biopsy	79
MGH830-1	Biopsy	92
MGH902-1	Biopsy	57
MGH908-2	Fluid	374
MGH920-1	Fluid	31
Average		81

Supplemental Figures

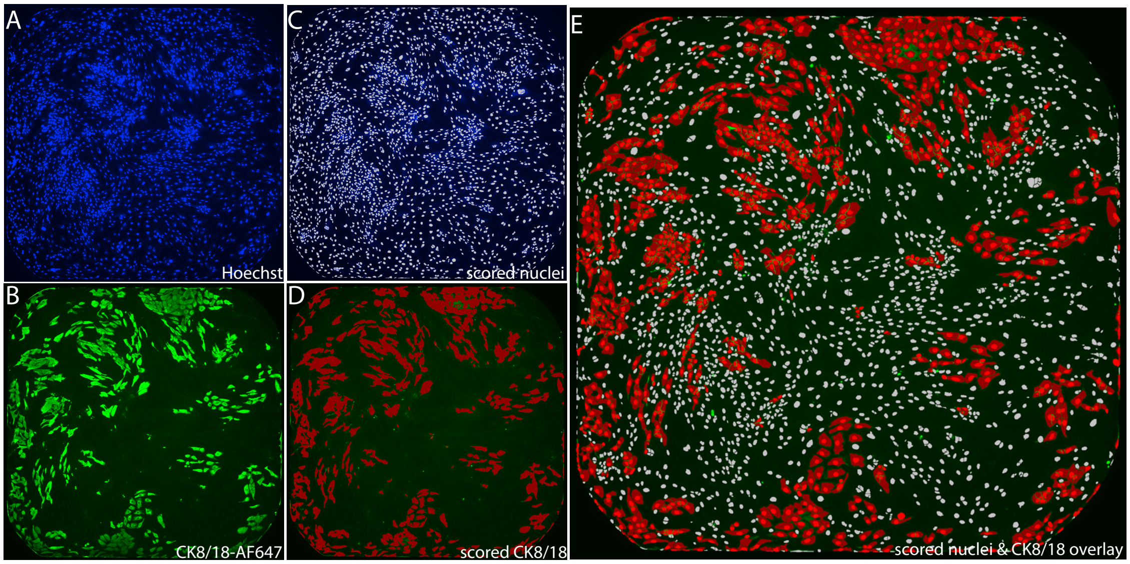

Figure S1. MetaExpress Cell Scoring Image Analysis of Hoechst- and CK8/18-Alexa647-Stained Primary Lung Tumor Cultures. Related to Figures 1-5. MGH721-1 biopsy culture was grown in a single well of a 384-well plate, fixed and stained with the rabbit anti-CK8/18 antibody followed by an Alexa Fluor-647-conjugated anti-rabbit IgG (B, green) and Hoechst 33342 (A, blue) as described in the Materials and Methods. Images were taken with Molecular Devices' Image Express Micro. Molecular Devices' MetaExpress software was used to score Hoechst-positive nuclei (C, white) and CK8/18-positive cytoplasm (D, red). The composite image of CK8/18-positive nuclei (red) and CK8/18-negative nuclei (white) is shown in E.

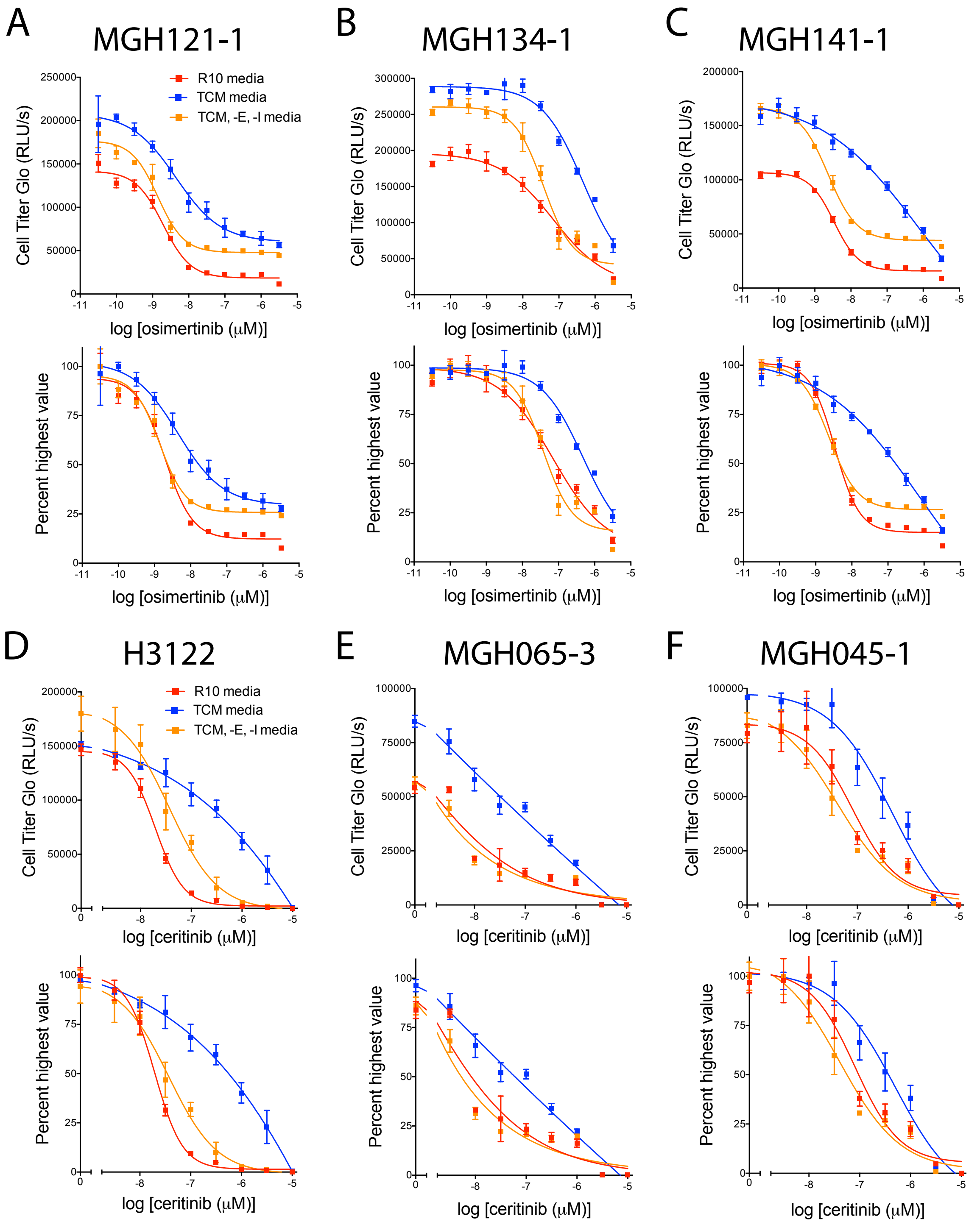

Figure S2. Sensitivity of *EGFR*-mutant (A-C) and *ALK*-translocated (D-F) lung cancer cells treated with EGFR or ALK inhibitors, respectively, growing in R10, TCM or TCM, -E, -I media. Related to Figure 2. Patient-derived *EGFR*-mutant (A-C) or *ALK*-translocated (B-C) lung cancer cells growing in R10 media (red curves), TCM media (blue curves) or TCM, -E, -I media (orange curves) in 384-well plates were treated with nine doses of the EGFR inhibitor osimertinib or the ALK inhibitor ceritinib for 4-7 days. Cell Titer-Glo reagent was used to measure cell viability. Raw relative light units per second (RLU/s) are illustrated on the top graphs and normalized values (percent highest) are depicted on the bottom. Data are represented as mean ± SD with n=4 replicate wells.

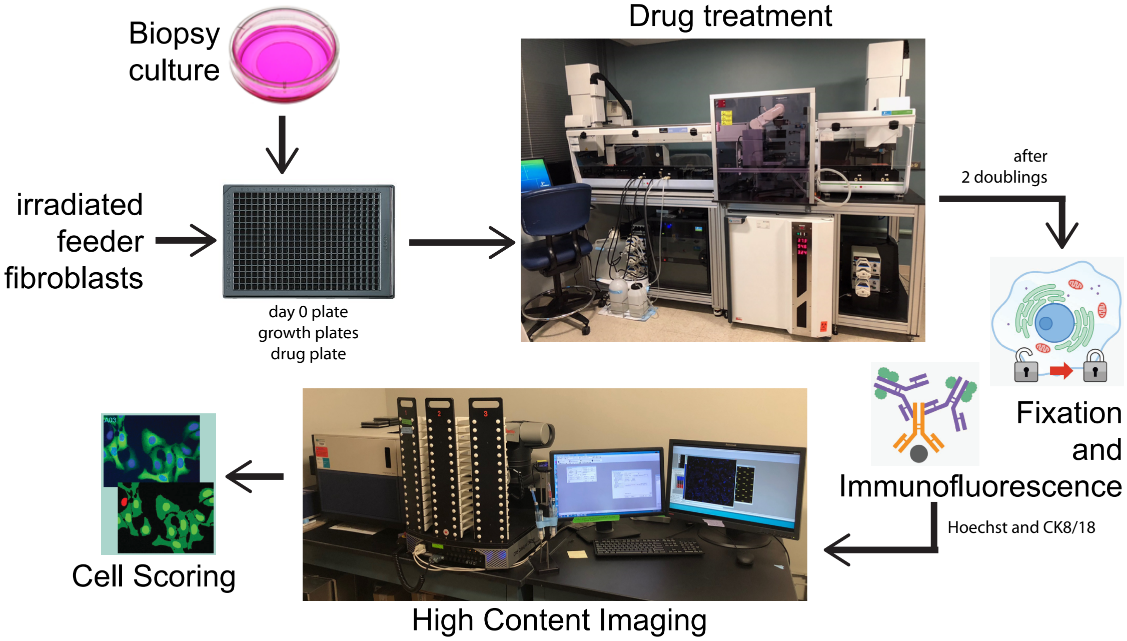

Figure S3. Schematic of the FAsT workflow. Related to Figures 3-5. Prior to plating early biopsy cultures, a monolayer of irradiated feeder fibroblasts was established in 384-well plates. Patient-derived culture plates included day 0 (fix at day of treatment initiation), a drug plate and growth plates. The drug plate consisted of a 10-dose treatment of both the targeted therapy of resistance, the subsequent prescribed targeted therapy, and a drug assumed to have no activity – for example, an ALK inhibitor in *EGFR*-mutant cancer cells, and vice-versa. Each dose entailed four wells. Growth plates allowed for the monitor of cells within the culture, and therefore determined when to stop the drug plate, preferable after two cancer cell doublings. After drug plate fixation, immunofluorescence was performed with the primary rabbit anti-CK8/18 antibody cocktail, secondary goat anti-rabbit IgG-AlexaFluor 684 antibody and Hoechst 33342. 384-well plates were imaged using Molecular Devices' Image Xpress Micro high content imager, and cell scoring was accomplished with their MetaExpress software.

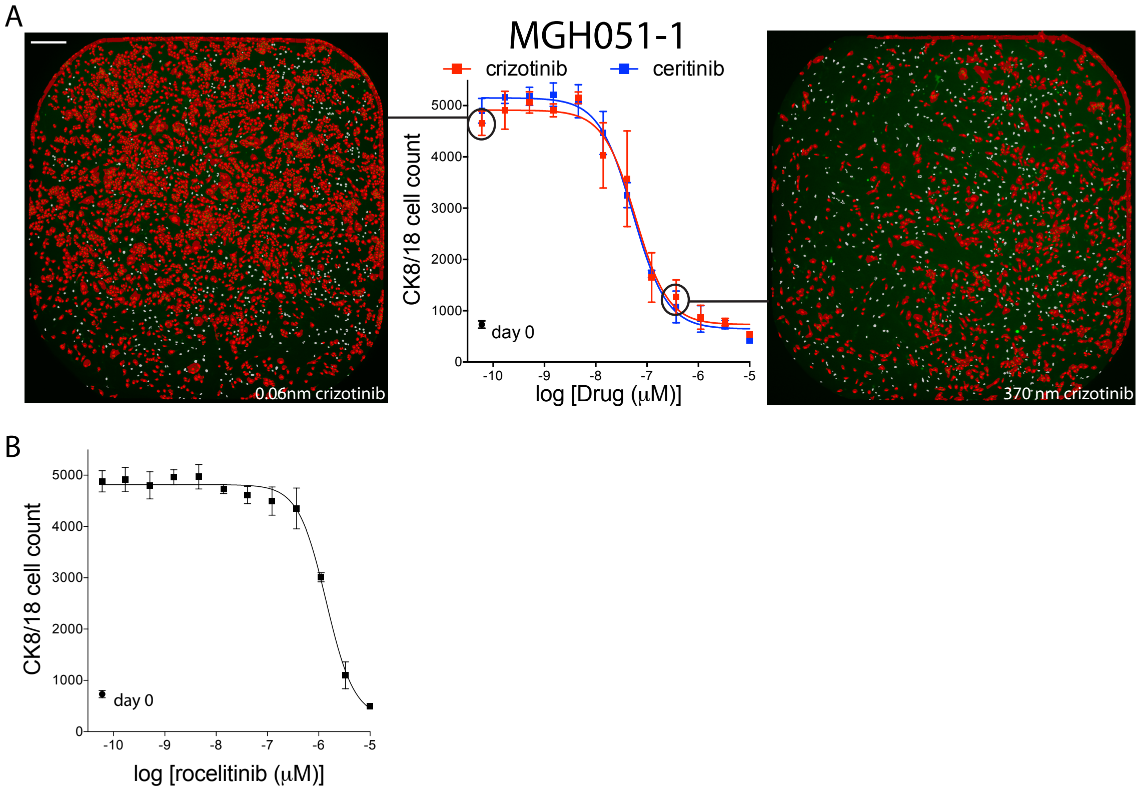

Figure S4. Response of *ALK*-mutant biopsy culture MGH051-1 to ALK inhibitors (A) or an EGFR inhibitor. Related to Figure 5. The early biopsy culture of *ALK*-translocated lung cancer MGH051-1 was plated on a monolayer of ~500 irradiated feeder fibroblasts in TCM, -E, -I media in 384-well plates, and treated with twelve doses of the ALK inhibitors crizotinib or ceritinib (A), or the EGFR inhibitor rociletinib (B) for 6 days. Plates were fixed and stained with Hoechst 33342 and the anti-CK8/18 antibody to determine the change in CK8/18-positive cell number. Black circles indicate cell number at day of treatment initiation (day 0). Representative images of a low and high dose of crizotinib are shown. Data are represented as mean ± SD with n=4 replicate wells. Scale bar is 270 μm.
